# The Ratio of 2-AG to Its Isomer 1-AG as an Intrinsic Fine Tuning Mechanism of CB1 Receptor Activation

**DOI:** 10.3389/fncel.2017.00039

**Published:** 2017-02-20

**Authors:** Klaudia Dócs, Zoltán Mészár, Sándor Gonda, Attila Kiss-Szikszai, Krisztina Holló, Miklós Antal, Zoltán Hegyi

**Affiliations:** ^1^Department of Anatomy, Histology and Embryology, Faculty of Medicine, University of DebrecenDebrecen, Hungary; ^2^Department of Botany, Faculty of Sciences, University of DebrecenDebrecen, Hungary; ^3^Department of Organic Chemistry, Faculty of Sciences, University of DebrecenDebrecen, Hungary

**Keywords:** 2-AG, 1-AG, cannabinoid, CB1, modulation, calcium signaling

## Abstract

Endocannabinoids are pleiotropic lipid messengers that play pro-homeostatic role in cellular physiology by strongly influencing intracellular Ca^2+^ concentration through the activation of cannabinoid receptors. One of the best-known endocannabinoid ‘2-AG’ is chemically unstable in aqueous solutions, thus its molecular rearrangement, resulting in the formation of 1-AG, may influence 2-AG-mediated signaling depending on the relative concentration and potency of the two isomers. To predict whether this molecular rearrangement may be relevant in physiological processes and in experiments with 2-AG, here we studied if isomerization of 2-AG has an impact on 2-AG-induced, CB1-mediated Ca^2+^ signaling *in vitro*. We found that the isomerization-dependent drop in effective 2-AG concentration caused only a weak diminution of Ca^2+^ signaling in CB1 transfected COS7 cells. We also found that 1-AG induces Ca^2+^ transients through the activation of CB1, but its working concentration is threefold higher than that of 2-AG. Decreasing the concentration of 2-AG in parallel to the prevention of 1-AG formation by rapid preparation of 2-AG solutions, caused a significant diminution of Ca^2+^ signals. However, various mixtures of the two isomers in a fix total concentration – mimicking the process of isomerization over time – attenuated the drop in 2-AG potency, resulting in a minor decrease in CB1 mediated Ca^2+^ transients. Our results indicate that release of 2-AG into aqueous medium is accompanied by its isomerization, resulting in a drop of 2-AG concentration and simultaneous formation of the similarly bioactive isomer 1-AG. Thus, the relative concentration of the two isomers with different potency and efficacy may influence CB1 activation and the consequent biological responses. In addition, our results suggest that 1-AG may play role in stabilizing the strength of cannabinoid signal in case of prolonged 2-AG dependent cannabinoid mechanisms.

## Introduction

Recognized as ubiquitous regulators of several physiological and pathological processes, endogenous cannabinoids control various fundamental biological phenomena from cell proliferation and metabolism to immune response and neurotransmission (Katona and Freund, 2012; Galve-Roperh et al., 2013; Hebert-Chatelain et al., 2014; Cabral et al., 2015; Maccarrone et al., 2015; Mazier et al., 2015). Importantly, cannabinoids exert their functions in the nervous system by modulating both excitatory and inhibitors neurotransmission, influencing a plethora of neurobiological phenomena from pain processing and motor coordination to anxiety and memory consolidation (D’Amico et al., 2004; Cannizzaro et al., 2006; Kano et al., 2009; Katona and Freund, 2012). The endocannabinoid system is usually described as a surprisingly diverse molecular toolbox, consisting of cannabinoid receptor type 1 and 2 (CB1 and CB2), their lipid-derived endogenous ligands known as endocannabinoids, and enzymes responsible for biosynthesis and degradation of endocannabinoids. Moreover, release of endocannabinoids not only activates CB1 and CB2 cannabinoid receptors (Andó et al., 2012), but also leads to the formation of various bioactive oxidative metabolites which broaden the spectrum of biological actions associated with endocannabinoid mobilization (Rouzer and Marnett, 2011). The best-characterized endocannabinoids, anandamide and 2-arachidonoyl-*sn*-glycerol (2-AG) share several common features. Both ligands are synthesized on demand from lipid precursors found in cell membranes, typically upon G-protein coupled receptor activation or depolarization, and released into the extracellular space (Kano et al., 2009). Anandamide and 2-AG target both CB1 and CB2 receptors (Pertwee, 2006) and influence cytosolic Ca^2+^ concentration (De Petrocellis and Di Marzo, 2009), and their effects are terminated primarily by enzymatic degradation including hydrolytic and also oxidative routes (Muccioli, 2010). However, the mechanisms decreasing the concentration of released 2-AG and anandamide are characteristically distinct. Although anandamide is stable chemically in organic solvents and aqueous solutions, 2-AG is prone to molecular rearrangement in water based media, i.e., the arachidonyl moiety moves from the 2-position to the 1-position of glycerol. This non-enzymatic isomerization, known as acyl migration, results in the formation of 1-arachidonoyl-*sn*-glycerol (1-AG), which is thermodynamically more stable than 2-AG, and the reaction proceeds until it reaches an equilibrium at 1:9 ratio of 2-AG and 1-AG (Martin, 1953). Still, in most biological systems, 2-AG mediated signaling is currently thought to be terminated by one or more degradative enzymes, such as monoacylglycerol lipase (MGL), fatty acid amide hydrolase (FAAH), α/β hydrolase domain 6 and 12 (ABHD6, ABHD12), cyclooxygenase-2 and also lipoxygenases (Muccioli, 2010; Savinainen et al., 2012). Depending on tissue and cell type, participation of these enzymes in the elimination of 1-AG and 2-AG is highly variable. In the central nervous system, for instance, MGL is responsible for approximately 85% of 2-AG catabolism (Dinh et al., 2002, 2004; Blankman et al., 2007), whereas in macrophages, ABHD12 may play a more prominent role as a 2-AG degrading enzyme (Fiskerstrand et al., 2010; Savinainen et al., 2012). Moreover, elimination of 1-AG and 2-AG is further diversified by the substrate preference of hydrolases. MGL has been demonstrated to equally accept and degrade both 1-AG and 2-AG, whereas ABHD6 and ABHD12 show preference to 1-AG over 2-AG (Navia-Paldanius et al., 2012). Thus, when 2-AG is released into aqueous extracellular space, the elimination rate is presumably influenced mostly by enzymatic processes and partly by acyl migration. Interestingly, however, 2-AG degrading enzymes are not necessarily found in the proximity of cannabinoid receptors, which is well illustrated in the superficial spinal dorsal horn, where most CB1 receptor-carrying axon terminals and glial processes lack MGL (Hegyi et al., 2009; Horváth et al., 2014; Dócs et al., 2015). Such molecular arrangement may provide opportunity for acyl migration to exceed enzymatic degradation, yielding biologically significant increasing concentration of 1-AG over time. Since it influences a plethora of physiological processes, 2-AG mediated intercellular signaling has extensively been investigated, including quantitative analysis of tissue levels of 2-AG, which is highly dependent on cell and tissue type, sample preparation and experimental settings. Interpretation also influence 2-AG quantities found in different papers, since 2-AG amounts are frequently expressed as the summed values for both 1- and 2-isomers, because isomerization was also identified as a post-isolation artifact (Ferrer et al., 2003; Van Sickle et al., 2005; Suplita et al., 2006). This may explain, that in the brain, for instance, reported tissue level of 2-AG varies from 0.23 to 65 nmol/g tissue (Sugiura et al., 2006).

To complicate matters further, several investigators found that 1-AG is a biologically active compound acting on CB1 receptor and increasing intracellular Ca^2+^ concentration (Sugiura et al., 1999), and it also activates TRPV1 (Zygmunt et al., 2013). Activity of 2-AG in cerebellar membrane preparations was also proposed to consist of activity of both 1- and 2-isomers (Savinainen et al., 2001). Thus, these findings suggest that isomerization yields in another biologically active compound which targets the same receptors as its precursor. This mechanism is not unusual in the endocannabinoid system, since oxidative and hydrolytic end-products of endocannabinoid catabolism are also bioactive (Rouzer and Marnett, 2011). Thus, acyl migration and consequent 1-AG formation might have an impact on endocannabinoid signaling, since the two isomers will most likely compete for ligand binding sites of CB1 receptors, which would represent an unusual mechanism, where the strength of net cannabinoid signal depends on the relative concentration and potency of the two isomers. Importantly, isomerization may or may not influence cannabinoid-dependent mechanisms depending on the half-life of acyl migration and the time-frame of the biological phenomenon. Prolonged CB1-mediated processes, such as an endocannabinoid tone can most probably be influenced by isomerization even if it has longer half-life. However, to influence rapid cannabinoid actions, like depolarization-induced suppression of excitatory or inhibitory synaptic transmission (DSE, DSI), acyl migration should proceed with a half-life that has never described earlier.

Here we demonstrate that rearrangement of 2-AG into its isomer 1-AG in water-based media is a moderately fast process, but it indeed yields another biologically active compound that exerts its effect also through the activation of CB1 receptors. We also show that, depending on the ratio of the two isomers, 1-AG may either act as a weak competitive antagonist of 2-AG, or its effect can also be additive to that of 2-AG maintaining CB1 receptor activation.

## Materials and Methods

### Decomposition of 2-AG in Aqueous Solutions

The endocannabinoid 2-AG and 1-AG were prepared and extracted as described earlier (Higuchi et al., 2010; Zhang et al., 2010; Zoerner et al., 2012) with some modifications. Briefly, 250 μl HBSS containing 0.625 μg/mL anandamide was spiked with either 2-AG (Cayman Chemical) or 1-AG (Cayman Chemical) dissolved in acetonitrile in a final concentration of 0.25 μg/mL) in test tubes, and incubated at 37°C for 1.25, 2.5, 5, or 10 min. Thereafter, the samples were frozen in liquid nitrogen and kept there until further processing. Zero time samples were spiked with 2-AG after freezing in liquid nitrogen. During sample preparation, 10 μl trifluoroacetic acid and 1000 μl hexane was added to the frozen samples, which were allowed to melt during constant vigorous shaking at 1400 rpm. This allowed the endocannabinoids to be transferred to the organic phase immediately after melting. TFA was added to stop the base-catalyzed isomerization. Then, the phases were separated by centrifugation at 13000 rpm for a minute. An 800 μl aliquot of the hexane layer was evaporated to dryness, redissolved in 40 μl acetonitrile containing 0.1% formic acid. Then, 20 μl of this solution was injected into the LC/MS. The experiment was performed in three replicates for each time point.

### Quantification of 1-AG and 2-AG with LC-MS

Changes in the amounts and ratio of 2-AG and 1-AG were determined on a YMC-Triart C18 (100 mm × 3.0 mm, 1.9 μm, 12 nm, YMC Co., Ltd, Kyoto 600-8106, Japan) column, using an Accela HPLC system (Thermo Electron Corp., San Jose, CA, USA) eluted with a gradient of acetonitrile (A) and water (B) containing 0.1%(V/V) formic acid each. The gradient was from 60% of A (hold for 2 min) to 90% A over 7 min, hold for 6 min and return to initial conditions and hold for 5 min to equilibrate the column. The LC system was coupled with a Thermo LTQ XL mass spectrometer (Thermo Electron Corp., San Jose, CA, USA) using positive-ion ESI mode as a method of ionization. The ion injection time was set to 100 ms. ESI parameters were as follows: spray voltage: 5 kV, source heater temperature: 280°C, capillary temperature of: 300°C, sheath gas flow: 25 units N_2_, auxiliary gas flow: 8 units N_2_. The tray temperature was set to 12°C and the column oven was set to 30°C to perform the optimal retention of the compounds in the reaction mixtures.

MS^2^ product-ion scans were obtained after collision-induced dissociation with helium as the target gas. Compound identification was based on their retention times (*t*_R_), HESI mass spectra and MS^2^ with authentic compounds as references. 1-AG and 2-AG levels were determined by LC-ESI-MS/MS in SRM mode and calibration with solutions of known concentrations of the analytes extracted for analyses. As an internal standard, anandamide was added before each sample extraction. SRM transitions were 379–287 for 1AG and 2AG and 348–287 for ANA, respectively. For all analytes of interest, recovery was calculated to be above 85% which is comparable to that published by Zoerner et al. (2012).

### Plasmid Construction

The mammalian expression vector pcDNA3 CB1 was used to overexpress CB1 receptor in COS7 cell line [generous gift from Mary Abood, Addgene plasmid # 13391, (Abood et al., 1997)]. To verify the successful transfection, the red fluorescent protein (RFP) coding expression vector CMV-Brainbow-1.0 H (a gift from Joshua Sanes, Addgene plasmid # 18720) was co-expressed in the cells under the control of the same CMV promoter as the pcDNA3-CB1 plasmid (Livet et al., 2007).

### Cell Culture and Transfection

COS7 cells (originated from ATCC, kindly provided by the Department of Biophysics, University of Debrecen) were grown to ∼90% confluence (10^4^ cell/cm^2^) in DMEM (Gibco, USA) supplemented with 10% fetal bovine serum (Sigma-Aldrich, USA), 100 U/ml penicillin, 100 mg/ml streptomycin and 2 mM glutamine (Gibco, USA) under 5% CO_2_ at 37°C. Prior to electroporation, cells were detached from 75 cm^2^ culturing flasks by incubation with Trypsin-EDTA (Sigma-Aldrich, USA), pelleted by centrifugation (900 × *g* for 10 min) and resuspended in DMEM (1.9 × 10^5^ cell/ml). This suspension was used for CB1/RFP co-transfection (20 μg/ml CB1 plasmid and 4 μg/ml RFP plasmid) and for control RFP transfection (4 μg/ml RFP plasmid). Electroporation was carried out by ECM830 electroporator (BTX, Harvard Apparatus, USA) using disposable 2 mm Gap Cuvette (Model No. 620, BTX, Harvard Apparatus, USA). The electroporation protocol was as follows: 220 V, two 500 μs width pulses with 1 s intervals.

### Ca^2+^ Measurements

Before Ca imaging experiments, transfected COS7cells were loaded with 1 μM Fluo-8-AM in the presence of 0.01% pluronic at room temperature for 30 min. Ca^2+^ imaging was carried out with an Andor Zyla 5.5 sCMOS camera attached to a differential spinning disk (DSD2, Andor Technology) built on an Olympus IX-81 inverse microscope. Using a 10× objective (NA:0.25), images of 540 pixels × 306 pixels (corresponding to 1400 mm × 790 mm field of view, which contained around 200 to 250 cells) were acquired at 15 frames per second with Andor iQ3 software. Fluo-8 filled cells were excited at 488 nm and emission was collected at 520 nm. Acquisition parameters (illumination intensity, exposure time, readout time, frame rate) were identical for all experiments. Changes in fluorescence intensities were measured over the entire COS7 cell surface by drawing freehand ROIs around single transfected COS7 cells with sharp RFP signal, that identified unequivocally the entire contour of the transfected cell. Ca^2+^ variations were estimated as changes of the fluorescence signal over baseline (Δ*F*/*F*_0_, where *F*_0_ was the average initial fluorescence). A region of interest was considered to respond to the application of a compound if Δ*F*/*F*_0_ three times the standard deviation of the baseline for at least five consecutive images. Experimental data were analyzed with Microsoft Excel 2013 (Microsoft), and FFT filtering to reduce noise and calculation of area under the curve (AUC) were performed with Origin Pro 8.0 (Originlab, Northampton, MA, USA). Statistical analysis was performed with two-tailed non-parametric Mann–Whitney *U* test. The differences were considered significant when the *p* level was < 0.05.

### SDS PAGE and Western Blot

The COS7 samples were sonicated in 20 mM TRIS (pH 7.4) lysis buffer supplemented with protease inhibitors (4 mM EDTA, 2.5 mM EGTA, 2 mM PMSF, 26 μM benzamidine, 8 μM pepstatin A, 2 μg/ml soybean trypsin inhibitor, 2 μg/ml leupeptin, 2 μg/ml aprotinin). The cell debris was removed by centrifugation (10 min at 1500 *g* and 4°C), then the supernatant was again centrifuged (20 min at 12,000 *g* and 4°C). The pellet was resuspended in lysis buffer containing 1% TRITON X-100 and 0.1% SDS. The samples were stored at -70°C until use.

The protein concentration of the samples was determined using the detergent compatible BCA assay (Pierce, Rockford, IL, USA). The samples were dissolved in reducing sample buffer (50 μg protein/lane) and run on 10% SDS-polyacrylamide gels (Laemmli, 1970). The separated proteins were electrophoretically transferred onto PVDF membrane (Millipore, Bedford, MA, USA).

The membranes were blocked with 10% bovine serum albumin (Sigma) in TTBS solution (20 mM TRIS, 500 mM NaCl, pH 7.5, 0.05% Tween-20). Membranes were incubated with anti- CB1antibody (1:1000, Cayman Chemicals, Ann Arbor, MI, USA, Cat. No: 10006590) for 2 h at room temperature. After extensive washing with TTBS the membranes were incubated with anti-rabbit IgG-HRP secondary antibody (DakoCytomation, Glostrup, Denmark). The labeled protein bands were visualized with 3, 3′-diaminobenzidine (Sigma).

### Immunocytochemistry

Transfected COS7 cells were grown on 24-well glass bottom plate and fixed with 4% paraformaldehyde dissolved in 0.1 M phosphate buffer for 10 min. Cells were then treated with 10% normal goat serum for 30 min, followed by an incubation with anti-CB1 antibody (1:2000, Cayman Chemicals, Ann Arbor, MI, USA, Cat. No: 10006590) for 2 h. After washing, goat anti-rabbit IgG secondary antibody conjugated with Alexa Fluor 488 (1:1000, Invitrogen) was applied for 1 h, then cells were covered with Vectashield-DAPI. Antibodies were diluted in PBS (pH 7.4) containing 1% normal goat serum. All incubation steps were carried out at room temperature.

Imaging of immunostained COS7 cells was carried out with an Olympus IX-81 inverse microscope attached to a DSD2 an Andor Zyla 5.5 sCMOS camera. Images were acquired using a 60× PlanApo N oil-immersion objective (NA: 1.40) and selecting the “high signal” disk of DSD2, and processed with Adobe Photoshop CS6.

The specificity of anti-CB1 antibody has extensively been characterized earlier in our laboratory (Hegyi et al., 2009). To test the specificity of the immunostaining protocol, transfected COS7 were incubated according to the immunostaining protocol described above with primary antibodies omitted or replaced with 1% normal goat serum. No immunostaining was observed under these conditions.

## Results

### 2-AG Rearranges Rapidly to 1-AG in HBSS

**FIGURE 1:**
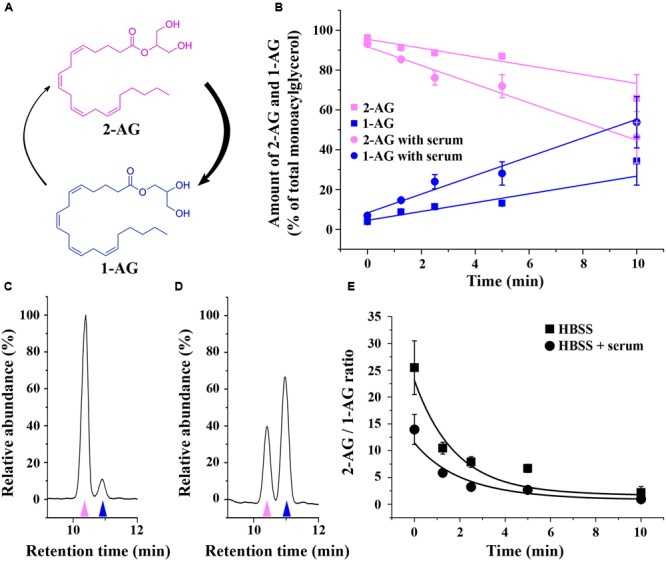
**Isomerization of 2-AG in HBSS. (A)** Structure and isomerization of 2-AG and 1-AG. **(B)** Changes in the amounts of 2-AG (magenta) and 1-AG (blue) over time in HBSS with (circles) or without (squares) serum. **(C–D)** Representative chromatograms illustrating the separation of 2-AG (magenta arrowhead) and 1-AG (blue arrowhead) in HBSS containing serum in the 0th min **(C)** and 10th min **(D)** of isomerization. **(E)** Change in the ratio of 2-AG and 1-AG due to acyl migration over time in HBSS with (circles) or without (squares) serum.

Many studies reported rearrangement of 2-AG into 1-AG (**Figure 1A**) under ambient conditions. In order to simulate the conditions of our *in vitro* experiments to the highest possible extent, Hank’s Balanced Salt Solution (HBSS), a commonly used cell medium (HBSS) was spiked with 2-AG and the decomposition curve at 37°C was determined using LC-MS. The other well-known endocannabinoid anandamide, which remains stable during the analytical procedure (Zoerner et al., 2012), was successfully used as internal standard to manage inaccuracies of liquid–liquid extraction and injections. The peak of anandamide showed no decrease over time.

In accordance with other publications employing RPMI medium (Rouzer et al., 2002), we found that 2-AG is rapidly converted to 1-AG also in HBSS (**Figure 1B**). The half-life of conversion was 16.16 ± 3.74 min (without serum, extrapolated) and 8.81 ± 2.51 min (with serum), which is within the same order of magnitude as reported for other physiological solutions. Differences may come from the differences in the media, and from the different initial concentration of 2-AG, since Rouzer et al. (2002) used 2 μg/mL (equal to 5 μM), while in our experimental settings the concentration was close to the order of magnitude used for the treatment of the cells (0.6 mM). The latter is comparable to the concentration of the OH^-^ present, possibly leading to altered kinetics of the reaction. The ratio of the two peaks is significantly changed within 2 min (**Figures 1C–E**). We have to add, however, that our experiments were carried out in pure physiological solution, free of cells or biological membranes, which helped us to significantly reduce the time frame of sample preparation and concomitant artifactual isomerization. This approach is different from those publications, in which 1-AG and 2-AG were isolated from biological samples requiring lengthy purification steps that provides possibility for post-isolation artifacts (Ferrer et al., 2003; Suplita et al., 2006).

Importantly, acyl migration takes place in any protic solvent (including water) and is actually catalyzed by OH^-^ present in all aqueous solutions at physiological pH (Rouzer et al., 2002). Therefore, we assume that 2-AG isomerizes to 1-AG on the minute timescale in any water-based media including the one used in the present study. In addition, rearrangement of 2-AG is accelerated in the presence of protein, that is likely to act as a catalytic surface that increases the reaction speed. Since actually all intracellular or extracellular fluids of the living organisms contain proteins, acyl migration is likely to be of significance *in vivo* as well.

### Overexpression of CB1 Receptor in COS7 Cells

**FIGURE 2:**
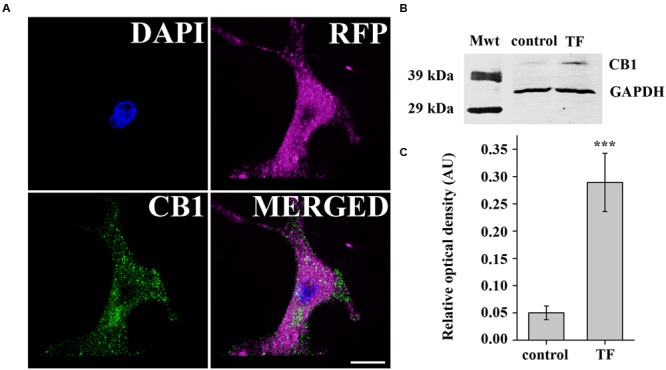
**Verification of CB1 receptor expression in transiently transfected COS7 cells. (A)** Immunofluorescent staining against CB1 receptor demonstrates strong expression of CB1 in transfected COS7 cells co-expressing RFP for easy identification. **(B)** Western blot analysis of CB1 receptor expression in transfected (TF) and also in native (control) COS7 cells. **(C)** Densitometry showed a fivefold increase in CB1 expression in transfected COS7 cells.

First, we aimed to establish a simple *in vitro* model expressing CB1 to study the cellular effects of 2-AG and 1-AG. Thus, we transiently transfected COS7 cells with pcDNA3 CB1 by electroporation after 5–7 passages to achieve overexpression of CB1 receptor, and verified the expression of CB1 by immunocytochemistry. CB1 immunostained puncta could be observed in high densities along the cell membrane and also in the cytoplasm of the transfected cells, identified by their RFP expression (**Figure 2A**). We also quantified CB1 protein levels 3 days after transfection. Western blot analysis with an anti-CB1 antibody confirmed the expression of CB1 in COS7 cells (**Figure 2B**), and found an approximately 5.6-fold increase in CB1 expression in the transfected cells (**Figure 2C**).

### Despite Its Conversion into 1-AG, the Biological Effects of 2-AG at Cannabinoid Receptors Are Barely Affected on the Minute Timescale

**FIGURE 3:**
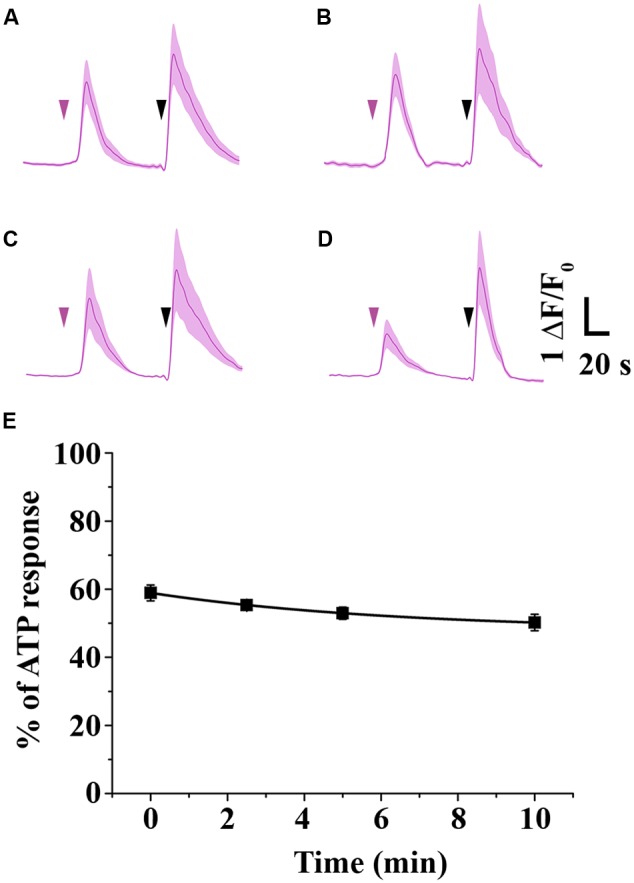
**Time-dependent change in the potency of 2-AG. (A–E)** Average Ca^2+^ transients in CB1 transfected COS7 cells induced by 1 mM 2-AG (magenta arrowhead), administered immediately **(A)**, 2.5 min **(B)**, 5 min **(C)** or 10 min **(D)** after dissolving the ligand in HBSS. **(E)** As isomerization proceeds, decreasing concentrations of 2-AG (see also **Figure 1B**) induce Ca^2+^ transients with only slightly diminished AUC values. Each data point represents an average of AUC values of Ca^2+^ transients evoked by 1 mM 2-AG, expressed as percentage of response to 180 mM ATP (black arrowhead).

Since 2-AG gradually disappears in HBSS due to isomerization, acyl migration may strongly influence the potency of 2-AG. Thus, we examined the effects of decreasing effective 2-AG concentration on CB1-induced Ca^2+^ transients by loading CB1 transfected COS7 cells with the fluorescent Ca^2+^ indicator Fluo-8-AM and monitoring Ca^2+^ signals evoked by administration of 2-AG. We identified all CB1-transfected cells in the visual field based on the RFP signal, and selected cells with sharp and distinct RFP-defined contour for analysis. To allow reliable comparison of the experimental data, we performed all Ca^2+^ imaging with identical settings and acquisition parameters. Changes in the fluorescence signal intensity over baseline (Δ*F*/*F*_0_) were plotted against time to illustrate alterations in the intracellular Ca^2+^ concentration. In the end of all Ca^2+^ measurement experiments, we applied 180 μM ATP as final treatment to verify viability of cells and obtain maximum Ca^2+^ response. Cells showing no response to ATP treatment were discarded from further analysis. Values of the AUC of Ca^2+^ transients evoked by ATP were considered as maximal responses for each cell. AUC values of cannabinoid-evoked Ca^2+^ signals were also calculated and normalized against responses to ATP by expressing CB1-dependent responses as percentage of maximal response. Cells in each well were treated only once to avoid desensitization of CB1 and allow registration of consistent responses. With these experimental settings, we treated the cells with 2-AG solution, that had a 1 μM starting concentration and was administered either immediately (“0-min experiment”), or incubated for 2.5, 5, and 10 min at room temperature before application. This amount of 2-AG is about 50% of the concentration that evokes maximal response, so any change in 2-AG levels will be approximately linearly reflected by changes is the Ca^2+^ transients. As shown in **Figure 3**, freshly prepared and immediately applied 2-AG induced robust transient-like elevation of intracellular Ca^2+^ concentration in CB1 transfected COS7 cells (**Figure 3A**; **Table 1**). Two and half minutes after preparation, 2-AG evoked Ca^2+^ signals with AUC values practically identical to that of 0-min experiment (**Figure 3B**; **Table 1**), and an additional incubation of 2-AG solution for 2.5 min caused only a marked diminution of Ca^2+^ responses (**Figure 3C**; **Table 1**). Application of 2-AG 10 min after preparation caused a weak and still not significant attenuation in cytosolic Ca^2+^ elevation (**Figure 3D**; **Table 1**). As we have shown, 2-AG is readily converted into 1-AG with a half-life of 8.8 min. However, the decrease in 2-AG concentration was not reflected in the evoked Ca^2+^ signals, as we detected only an insignificant minor decrease in biological responses.

**Table 1:** Time-dependent decrease in area under the curve (AUC) values of Ca^2+^ transients (expressed as percentage of response to 180 mM ATP) in response to 2-AG that was left in HBSS containing 10% serum to allow isomerization.

Time (min)	AUC (% of ATP response)	*n*
0	58.92 ± 2.326	150
2.5	55.3 ± 1.025	141
5	52.96 ± 1.777	139
10	50.22 ± 2.434	137

### 1-AG Concentration-Dependently Increases Intracellular Ca^2+^ Concentration by Activating Cannabinoid Receptors

**FIGURE 4:**
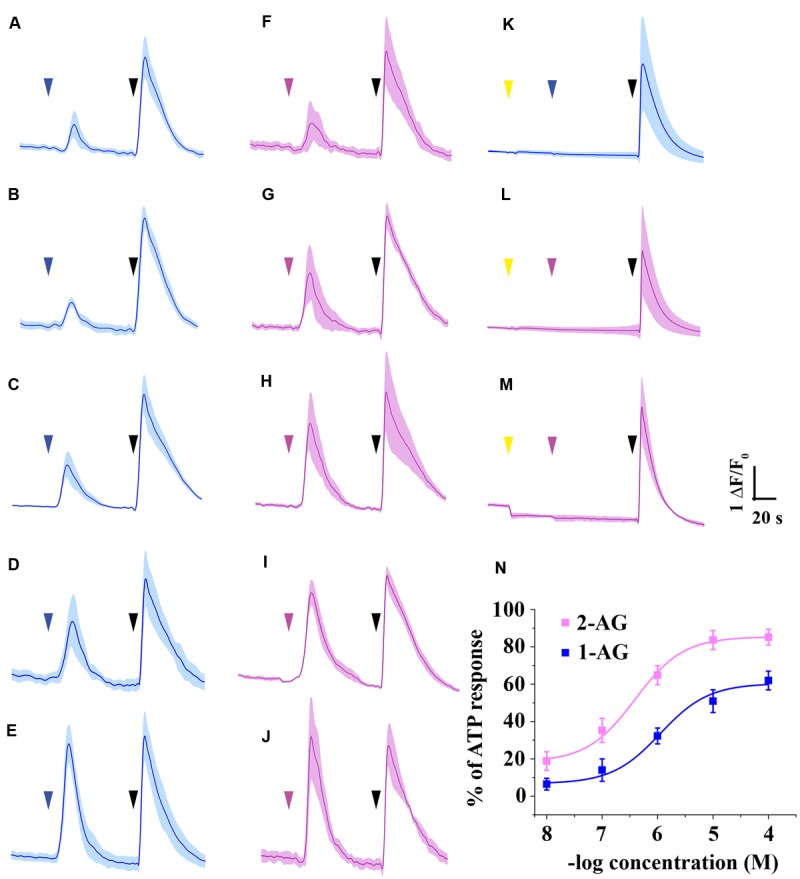
**Ca^2+^ transients induced by 1-AG and 2-AG. (A–E)** Average Ca^2+^ transients induced by 10^-8^ **(A)**, 10^-7^ **(B)**, 10^-6^ **(C)**, 10^-5^ **(D)**, or 10^-4^ M **(E)** 1-AG (blue arrowhead). **(F–J)** Population average of Ca^2+^ transients in response to application of 2-AG in 10^-8^ **(F)**, 10^-7^ **(G)**, 10^-6^ **(H)**, 10^-5^ **(I)**, or 10^-4^ M **(J)** (magenta arrowhead). **(K–L)** Application of AM251 in 5 μM (yellow arrowhead) prevents 10 μM 1-AG (**K**, blue arrowhead) and 1 μM 2-AG **(L**, magenta arrowhead) from evoking Ca^2+^ transients. **(M)** AM251 in 5 μM (yellow arrowhead) induced a drop in the baseline in case of several cells. 2-AG in 1 μM (magenta arrowhead) failed to evoke any biological response in these cells. **(N)** Standard concentration-response curves of 1-AG and 2-AG. Each data point represents an average of AUC value of Ca^2+^ transients induced by various concentrations of 1-AG (as shown in **A–E**, blue) or 2-AG (based on **F–J**, magenta), expressed as percentage of response to 180 μM ATP (black arrowheads).

As the arachidonoyl moiety moves from the 2-position to the 1-position of glycerol, not only the effective concentration of 2-AG decreases continuously, but also the amount of 1-AG increases gradually in aqueous solutions until the equilibrium between 2-AG and 1-AG is reached at approximately 1:9 ratio. Here we investigated if the 1-isomer interacts with CB1 in our experimental conditions, and compared its pharmacological properties to those of 2-AG. We administered 1-AG solution of various concentration spanning from 10 nM to 100 μM to CB1 transfected COS7 cells, and examined if the treatment induced any change in the intracellular Ca^2+^ concentration in our experimental conditions. As shown in **Figures 4A–E,N** and **Table 2**, starting at low micromolar concentration, 1-AG evoked Ca^2+^ transients with strictly concentration-dependent amplitude and AUC values.

**Table 2:** Area under the curve values of Ca^2+^ transients (expressed as percentage of response to 180 μM ATP) in response to various concentration of 1-AG and 2-AG.

Concentration	2-AG		1-AG	
	AUC (% of ATP response)	*n*	AUC (% of ATP response)	*n*
10 nM	18.81 ± 4.99	133	6.45 ± 3.12	129
100 nM	35.24 ± 6.45	137	13.98 ± 5.99	130
1 μM	64.84 ± 5.11	145	32.26 ± 4.24	134
10 μM	83.67 ± 5.12	160	50.91 ± 6.11	137
100 μM	85.19 ± 4.23	159	61.99 ± 5.01	144

To compare the potency and efficacy of the two isomers, we repeated the above experiment, now by applying various concentration of 2-AG spanning from 10 nM to 100 μM. In agreement with earlier observations (Sugiura et al., 1999), 2-AG also induced Ca^2+^ transients even in nanomolar concentration (**Figures 4F–J,N**). Concentration-response curve of 1- and 2-AG clearly demonstrates that potency of 2-AG (EC50 = 0.6 μM) is threefold higher than that of 1-AG (EC50 = 1.9 μM), and 2-AG was also found to be more efficacious. (**Figure 4N**; **Table 2**).

To demonstrate that 1- and 2-AG induce Ca^2+^ signals through a CB1 receptor mediated pathway, we applied the selective CB1 antagonist AM251. Effects of 1-AG were exclusively mediated by CB1 receptors, since application of AM251 prevented the raise in intracellular Ca^2+^ in response to 10 μM 1-AG in 99.37 ± 0.51% of the cells (**Figure 4K**).

Application of AM251 completely abolished 2-AG evoked Ca^2+^ transients also in the majority of the cells (**Figure 4L**), however, 2.33 ± 0.89% of COS7 cells still showed responses to 1 μM 2-AG. Preincubation with AM251 caused a pronounced decrease in, but could not fully prevent the 2-AG induced raise of, intracellular Ca^2+^ concentration in these cells (data not shown).

Preincubation of the cells with AM251 at 5 μM caused an apparent drop of the baseline in case of 6.16 ± 0.91 of the cells (**Figure 4M**). This may most probably be the result of the inhibition of a basal endocannabinoid tone by AM251, but, as an inverse agonist, it may also decrease constitutive CB1 receptor activity. Thus, it is very likely that the AM251-induced drop of the baseline is CB1-dependent, but we cannot exclude other mechanisms in the background of this phenomenon, such as the inhibition of adenosine A1 receptors by AM251.

### Accumulation of 1-AG Compensates the Decreasing 2-AG Concentration and Consequent Drop in Biological Responses

**FIGURE 5:**
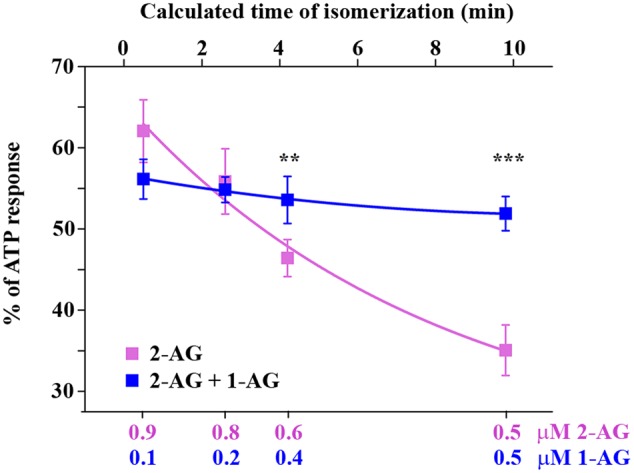
**CB1 receptor mediated intracellular Ca^2+^ increase in response to application of 2-AG alone and in combination with 1-AG.** Each data point represents an AUC value of Ca^2+^ transients expressed as percentage of response to 180 μM ATP. Decreasing concentrations of 2-AG induce diminished Ca^2+^ transients (magenta). This effect is compensated by increasing amounts of 1-AG, i.e., 1-AG at low concentration acts as competitive antagonist, but its effect turns to additive as its concentration increases (blue). The values on the top abscissa were calculated from the ratio of the artificial mixtures of the two isomers with the aid of **Figure 1E**. ^∗∗^*p* < 0.01; ^∗∗∗^*p* < 0.005.

Isomerization of 2-AG creates an interesting situation, since both 1- and 2-isomers of arachidonoylglycerol target CB1 and it is very likely that they will compete for the ligand binding sites of CB1. However, depending on the relative concentration of the two isomers with different potency and ligand binding sites available in the biological environment, combined effects of 1-AG and 2-AG may be either additive or antagonistic. We hypothesized that, as isomerization proceeds after the moment of 2-AG mobilization, the antagonistic effect of accumulating 1-AG becomes gradually more pronounced, resulting in the attenuation of 2-AG evoked biological responses. To investigate this possibility, we first calculated the change in ratio of the two isomers over 10 min. Based on our measurements (**Figure 1C**), the amounts of 2-AG and 1-AG change so that, 0.5, 2.6, 4.2, and 9.8 min after dissolving 2-AG in HBSS, their ratio is approximately 9:1, 8:2, 6:4, and 5:5, respectively. Thus, in this experiment we treated transfected COS7 either with 2-AG alone in a descending serial dilution (0.9, 0.8, 0.6, and 0.5 μM), or with a mixture containing increasing quantities of 1-AG (0.1, 0.2, 0.4, and 0.5 μM) complementing the decreasing concentration of 2-AG to mimic the progress of isomerization. As expected, the drop in effective 2-AG concentration was paralleled by a pronounced diminution of evoked Ca^2+^ transients (**Figure 5**; **Table 3**). Surprisingly, however, effects of 1-AG on 2-AG evoked Ca^2+^ transients were strongly dependent on the relative concentration of the two isomers. Although we observed a weak antagonistic effect of 0.1 μM 1-AG against 0.9 μM 2-AG, gradually increasing concentration of 1-AG proved to be additive and increased Ca^2+^ signals evoked by declining quantities of 2-AG. The slope of concentration-response curve of decreasing [2-AG] – increasing [1-AG] pairs mimicked surprisingly well the time-dependent minor and not significant changes of 2-AG induced Ca^2+^ responses (**Figure 5**; **Table 3**). Our measurements showed also that the effect of 1-AG on 2-AG dependent cellular responses is neutral at approximately at 0.8 μM 2-AG to 0.2 μM 1-AG ratio, that is reached in 2.3 min from the moment when 2-AG meets the aqueous medium (**Figure 5**).

**Table 3:** Area under the curve values of Ca^2+^ transients in response to 2-AG alone (magenta rows) and in combination with 1-AG (blue rows) (expressed as percentage of response to 180 μM ATP).

Concentration of		AUC (% of ATP response)	*n*
2-AG (μM)	1-AG (μM)		
0.9	0	62.05 ± 3.85	152
	0.1	56.13 ± 2.44	142
0.8	0	55.85 ± 4.02	150
	0.2	54.83 ± 1.56	144
0.5	0	46.41 ± 2.28	147
	0.5	53.57 ± 2.9	145
0.1	0	35.07 ± 3.12	145
	0.9	51.89 ± 2.12	148

## Discussion

Endogenous cannabinoids are ubiquitous intercellular messengers playing essential role in a variety of physiological and pathological processes. Although the endocannabinoid system has been extensively investigated in practically every type of mammalian tissue (Kano et al., 2009; Maccarrone et al., 2015), several questions and controversies remain to be solved (Piomelli, 2014). One of these issues involve the mechanisms that decreases the concentration and terminates the effects of the endocannabinoid 2-AG, since besides the enzymatic degradative pathways, 2-AG, as member of 2-monoglycerol family, is chemically unstable and prone to acyl migration which results in the formation of 1-AG (Martin, 1953). This molecular rearrangement generates difficulties when quantifying amounts of 2-AG from biological samples (Vogeser and Schelling, 2007; Astarita and Piomelli, 2009; Pastor et al., 2014) and interpreting the effects of 2-AG at cannabinoid and TRP receptors (Zygmunt et al., 2013). Here we investigated the kinetics of 2-AG isomerization into 1-AG and found that acyl migration is a rapid molecular rearrangement with a half-time of 16.16 min in HBSS and 8.8 min in HBSS containing 10% serum at 37°C. Although our measurements were carried out in cell-free physiological solutions, these results may indicate the biological relevance of 2-AG isomerization in certain cellular environments, since 2-AG degrading hydrolases decrease the amounts of 2-AG with a half-life of 19–28 min depending on the cell density (Di Marzo et al., 1999). In that case, acyl migration and 1-AG formation seems to be approximately two times faster than enzymatic inactivation of 2-AG. Importantly, isomerization does not have an impact on the findings of the cited paper, since 1- and 2-monoacyglycerols are equally accepted by the investigated hydrolases, and their catabolism results in the formation of the same end-products (Tornqvist and Belfrage, 1976; Di Marzo et al., 1999). In many other physiological conditions, however, where the half-life of 2-AG signaling is shorter than the rate of isomerization, formation of 1-AG is unlikely to play physiologically relevant role. This may be the case in 2-AG mediated retrograde synaptic transmission, where the half-lives of depolarization induced suppression of excitation of inhibition has been shown to fall in the 15–40 s range (Kano et al., 2009).

Earlier studies demonstrated, that isomerization of 2-AG depends primarily on the pH and partly on the ionic strength of the milieu, and found faster acyl migration with a half-life of 10 min in serum-free and 2.3 min in serum-supplemented RPMI medium at 37°C (Rouzer et al., 2002). Although we cannot fully explain this difference, we assume that the faster isomerization may be the result of the richer and well supplemented RPMI medium used in the cited study. The somewhat slower chemical transformation which we found still indicates that, regardless of the presence of monoacylglycerol degrading enzymes, release of 2-AG into aqueous extracellular space leads to the formation and possibly to the temporary accumulation of 1-AG, which gives special importance to bioactivity or inactivity of 1-AG at cannabinoid receptors.

Therefore, we next investigated if 1-AG has any CB1 mediated biological activity, and found that 1-AG transiently increases intracellular Ca^2+^ concentration in a dose dependent manner. This finding, in good agreement with earlier and frequently overlooked studies (Sugiura et al., 1999), demonstrates that 1-AG is indeed a bioactive molecule that activates CB1. The EC50 of 1-AG was found to be one order of magnitude higher than that of 2-AG, and 2-AG was more efficacious in producing biological responses in CB1 transfected COS7 cells. However, 1-AG can also be considered as a high efficacy agonist at CB1 receptor, and isomerization of 2-AG, therefore, does not represent a non-enzymatic inactivation mechanism, but yields another bioactive molecule activating the same receptors as its precursor. We have to add, however, that during physiological conditions, a cellular environment may never reach such a high concentration of 1-AG that evokes maximal response. Thus, 1-AG is likely to incompletely activate CB1 receptors.

**FIGURE 6:**
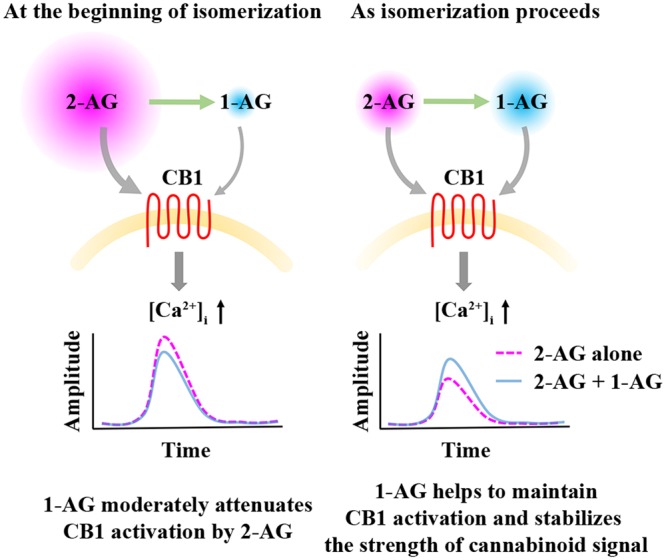
**Effects of 1-AG on 2-AG mediated CB1 receptor activation and consequent Ca^2+^ signaling.** At the beginning of isomerization **(left)**, 1-AG is present in low concentration and exerts a weak antagonistic effect against 2-AG at CB1. As isomerization continues **(right)**, 2-AG is gradually replaced by 1-AG, and the activation of CB1 by the increasing concentration of 1-AG becomes more pronounced. Thus, 1-AG can now compensate the decrease in the concentration of 2-AG and the consequent diminution of Ca^2+^ signals by maintaining CB1 activation.

Acyl migration following 2-AG release creates a rather unique situation, when the monoacylglycerol concentration is relatively constant, but the ratio of the two isomers changes rapidly as 2-AG is gradually replaced by 1-AG, and the two isomers may compete for ligand binding sites of CB1. Therefore, we next studied the biological consequences of this process by preparing mixtures of the two isomers in a ratio that simulates the time-dependent change in their relative quantities. The moderate and not significant diminution of effects of 2-AG on Ca^2+^ signaling over time was surprisingly similar to that of the appropriate artificial mixtures of the two isomers representing given time point of isomerization. Importantly, results of both experiments were significantly different from the more pronounced decline evoked by decreasing quantities of 2-AG alone indicating, that accumulating 1-AG stabilizes the cannabinoid signal and masks the biological consequences of isomerization-related drop in 2-AG concentration. This may explain why chemical instability of 2-AG did not have any obvious impact on experimental settings with 5–10 min or even longer time frames (Szabo et al., 2006; De Luca et al., 2014; Stanley and O’Sullivan, 2014; Griebel et al., 2015), and it is also very likely that such measurements result in data mirroring at least partly or mostly effects of 1-AG (**Figure 6**).

Bioactivity of 1-AG at CB1 and its ability to effectively compensate the rapid elimination of 2-AG (**Figure 6**) may represent an essential mechanism in maintaining long-lasting effects of 2-AG and also basal endocannabinoid tone. However, our results and interpretation have certain limitations. We carried out experiments on transfected cell cultures, which may be different from most *in vivo* conditions in terms of cell physiology and metabolism, intercellular connections and extracellular environment. For instance, various enzymes degrading monoacylglycerols may effectively modify the ratio of the two isomers. MGL equally accepts both 1-AG and 2-AG, whereas ABHD6 and ABHD12 show preference to 1-AG over the 2-isomer (Navia-Paldanius et al., 2012). Thus, differential expression of these hydrolases in various types of cells and tissues may result in highly different 1-AG/2-AG ratios. Moreover, 2-AG release into aqueous extracellular space is frequently mentioned as an important step of cannabinoid signaling, but its proper mechanism is poorly understood (Bisogno et al., 1997; Sugiura and Waku, 2000; Di Marzo et al., 2005), and is further complicated by lateral diffusion, i.e., 2-AG is dissolved in, and travels along cell membranes, which may delay or prohibit acyl migration (Makriyannis et al., 2005; Hurst et al., 2010). Thus, biological processes associated with 2-AG release may represent composite effects of various mixtures of the 1- and 2-isomers. Fast endocannabinoid signaling, such as homosynaptic retrograde neurotransmission is most likely dominated by 2-AG, whereas in case of prolonged effects of cannabinoids, like tonic cannabinoid receptor activation (Sagar et al., 2010), participation of 1-AG in stabilizing the net cannabinoid signal and maintaining CB1 activation may be more prominent. This latter possibility opens the question if prolonged incubation with the two isomers induce differential degrees of CB1 desensitization which may further diversify the outcome of isomerization.

Importantly, commercially available 2-AG preparations already contain approximately 10% 1-AG, therefore the isomerization process starts at 9:1 ratio. Although 1-AG masks the isomerization-associated drop in 2-AG concentration, the strength of the evoked biological responses will necessarily reflect the effects of various mixtures of the two isomers in only several minutes. Investigators should be aware of acyl migration in experiments employing 2-AG, and choose carefully a time frame short enough to prevent formation of 1-AG so that the results can reliably be associated with 2-AG.

## Author Contributions

KD conducted immunocytochemical and calcium imaging experiments and analyzed data. ZM designed and conducted electroporation and revised the manuscript. SG and AK-S designed and carried out LC-MS measurements and revised the manuscript. KH carried out Western blot analysis. MA and ZH conceived the project. ZH designed and performed immunocytochemical and calcium imaging experiments, analyzed data and wrote the manuscript.

## Conflict of Interest Statement

The authors declare thatthe research was conducted in the absence of any commercial or financial relationships that could be construed as a potential conflict of interest.

## Chemical compounds studied in this article

2-arachidonoyglycerol: (2-AG, PubChem CID: 5282280);
1-arachidonoyl-sn-glycerol: (1-AG, PubChem CID: 16019980);
AM251: (PubChem CID: 2125);
adenosine triphosphate: (ATP, PubChem CID: 5957)
